# Design of Heterogeneous Hoveyda–Grubbs Second-Generation Catalyst–Lipase Conjugates

**DOI:** 10.3390/molecules21121680

**Published:** 2016-12-06

**Authors:** Anthony Neville, Javier Iniesta, Jose M. Palomo

**Affiliations:** Department of Biocatalysis, Institute of Catalysis (CSIC), Marie Curie 2, Cantoblanco, Campus UAM, 28049 Madrid, Spain; c12301636@mydit.ie (A.N.), javier.iniesta@estudiante.uam.es (J.I.)

**Keywords:** heterogeneous catalyst, ruthenium catalyst, ring-closing metathesis, lipases, hybrid

## Abstract

Heterogeneous catalysts have been synthesized by the conjugation of Hoveyda–Grubbs second-generation catalyst with a lipase. The catalytic properties of the organometallic compound in solution were firstly optimized, evaluating the activity of Ru in the ring-closing metathesis of diethyldiallymalonate at 25 °C at different solvents and in the presence of different additives. The best result was found using tetrahydrofuran as a solvent. Some additives such as phenylboronic acid or polyetheneglycol slightly improved the activity of the Ru catalyst whereas others, such as pyridine or dipeptides affected it negatively. The organometallic compound immobilized on functionalized-surface materials activated with boronic acid or epoxy groups (around 50–60 µg per mg support) and showed 50% conversion at 24 h in the ring-closing metathesis. Cross-linked enzyme aggregates (CLEA’s) of the Hoveyda–Grubbs second-generation catalyst with *Candida antarctica* lipase (CAL-B) were prepared, although low Ru catalyst was found to be translated in low conversion. Therefore, a sol–gel preparation of the Hoveyda–Grubbs second-generation and CAL-B was performed. This catalyst exhibited good activity in the metathesis of diethyldiallymalonate in toluene and in aqueous media. Finally, a new sustainable approach was used by the conjugation lipase–Grubbs in solid phase in aqueous media. Two strategies were used: one using lipase previously covalently immobilized on an epoxy-Sepharose support (hydrophilic matrix) and then conjugated with grubbs; and in the second, the free lipase was incubated with organometallic in aqueous solution and then immobilized on epoxy-Sepharose. The different catalysts showed excellent conversion values in the ring-closing metathesis of diethyldiallymalonate in aqueous media at 25 °C.

## 1. Introduction

Chemical reactions catalyzed by enzymes represent the best environmentally friendly scenario in organic chemistry [1,2,3].

These catalysts work in very mild reaction conditions with exquisite selectivity in many cases. However, very few or no examples of the existence of synthetic enzymes in Nature, in particular the metathesis reaction, have been reported [4].

This limitation has been approached in the last years, producing the first artificial enzymes with activity in metathesis [5,6,7,8]. Different strategies, such as a combination of synthetic protein chemistry and molecular biology [5,6], dative coordination of an organometallic ligand to the metal in the protein scaffold [7] or by covalent incorporation with a specific group in the active site [8], have been employed.

Nevertheless, the possible scale up of the strategy to routine synthetic chemistry leads to the development of a strategy which considers the application of a heterogeneous system to be necessary.

As occurs with natural enzymes, the creation of heterogeneous catalysts is essential for their industrial implementation.

In this sense, the use of immobilization technology to specific support materials or the generation of hybrids systems using protein as a scaffold to conjugate with the corresponding transition metal catalysts has been a successful example to create heterogeneous artificial metalloenzymes [9,10,11,12]. In particular, the application of enzymes with high catalytic versatility could generate higher possibilities, depending on the transition metal or metal binding ligands inserted. Moreover, the accessibility to a high amount of protein is mandatory in order to obtain a high amount of final artificial metalloenzymes.

To this purpose, lipases—acylglycerol hydrolases—fulfill both requirements. These are very versatile enzymes with successful results in different processes using non-natural substrates [13] and they are commercially available in large amounts. Recently, these enzymes have been combined with metals in the preparation of several hybrids catalysts [14,15,16,17].

**Scheme 1:**
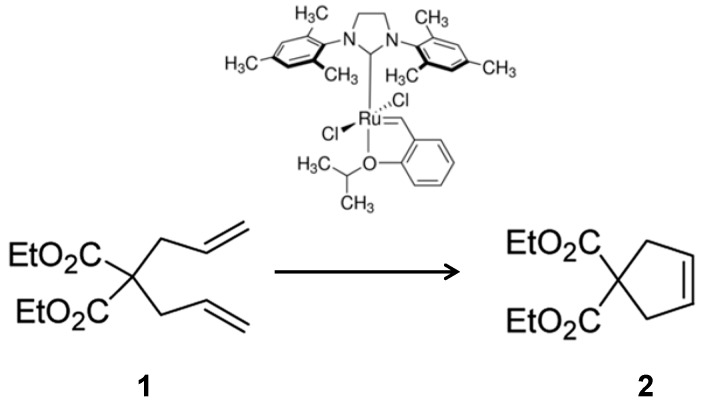
Ring-closing metathesis of diethyldiallylmalonate (**1**) catalyzed by Hoveyda–Grubbs second-generation catalyst.

Herein, different technologies to obtain a conjugate heterogeneous organometallic-protein system have been described. For that, the combination of lipase from *Candida antarctica* (fraction B) (a very robust and commercially available enzyme) and the Hoveyda–Grubbs second-generation catalyst has been used. The organometallic molecule was conjugated with the protein at different immobilization systems, such as enzyme aggregate, enzyme immobilized on support materials or by thesol–gel entrapment system. The catalytic capacity of the ruthenium organometallic complex in the different protein-conjugates was evaluated in the ring-closing metathesis (RCM), among the most important C–C bond forming reactions [18,19,20] of diethyldiallymalonate (**1**) (Scheme 1) at mild conditions.

## 2. Results and Discussion

### 2.1. Activity of Soluble Hoveyda–Grubbs Second-Generation Catalyst at Different Conditions

The reactivity of the *Hoveyda–Grubbs second-generation* catalyst in the ring-closing metathesis reaction of **1** (Scheme 1) was evaluated in the presence of different solvents at 25 °C. A full metathesis conversion was achieved in most of the solvents tested. Faster conversion was obtained in tetrahydrofuran (THF) (30 min) (maybe because THF could stabilize the active propagating Ru carbine [21]) and CH_2_Cl_2_ (1 h), whereas in others such as hexane, heptane, oluene, DMSO, acetonitrile or ethanol was necessary for incubation at 2 h (data not shown). In a mixture of 20% (*v*/*v*) acetonitrile in water, the catalyst was not reactive, and directly not soluble in pure water. A second parameter to consider is the effect of the presence of additives. Indeed, it has been demonstrated that the addition of several molecules during the metathesis reaction (such as acetic acid, benzoquinones, or thioureas) [22,23,24] positively affect the final catalytic properties of the organometallic compound. Different additives could result in completely different active species from the same Grubbs catalyst and trigger different catalytic pathways to produce structurally unique products.

In this way, here, the optimization of the metathesis using the homogeneous catalyst in the presence of additives of different nature in THF was evaluated (Table 1).

The addition of Lewis acids (BF_3_-Et_2_O, TMSOTf) to the reaction decreased the metathesis conversion catalyzed by soluble Grubbs catalyst between 25% and 30% (entries 2–3, Table 1). The presence of amines resulted in worse results, showing 40% activity adding pyridine (entry 5, Table 1). In this case, this could be because of the high vulnerability of the metallacyclobutane intermediate formed in the presence of ethylene, which is rapidly decomposed in the presence of a base, even at room temperature [25].

However, the use of different ionic liquids as additives slightly improved the activity of the catalyst.

The presence of different polyol polymers did not alter significantly the activity of the catalysts, being positive for their use in the creation of heterogeneous catalysts. Surprisingly, the most improvement of the catalytic performance of the organometallic compound was achieved using phenylboronic acid as additives (entry 14, Table 1).

Finally, aminoacids and peptides were tested as additives with different results. Aspartic acid dimethyl diester caused a decrease in the activity of the catalysts of 70% in the metathesis reaction, because of the presence of the free amine. However, a cysteine-containing *N*-acetylated short hydrophobic tripeptide (NHAc–Cys–Phe–Phe–CONH_2_) slightly improved the rates in the metathesis reaction. Using another more hydrophilic cysteine-containing peptide sequence, the effect was slightly negative.

Recently, the modification of ruthenium catalyst by the introduction of amino acids as ligands produced good results in activity and selectivity in ring-closing metathesis [26].

Thus, the procedure of activation by the corresponding silver salt of the dipeptide N-terminal protected with tert-butyloxycarbonyl protecting group (BOC), BOC–Ala–Gly–COOH was used. The silver salt was synthesized as previously described [26] and was incubated as an additive in a solution of Hoveyda–Grubbs catalyst in THF. In this case, unfortunately the new adduct exhibited 75% less activity of the non-modified one (entry 17, Table 1).

Therefore, with the addition of several additives such as ionic liquids, some polymers or peptides showed positive effects and clearly the catalytic reactivity of the Grubbs catalyst was strongly decreased in the presence of amine-containing molecules and by specific ligand modification with Boc-peptide.

### 2.2. Preparation of Heterogeneous Catalyst and Application in RCM

After the screening of the RCM conditions of the homogeneous Hoveyda–Grubbs second-generation catalyst, different strategies to generate a heterogeneous catalyst have been used.

Firstly, the Hoveyda–Grubbs second-generation catalyst was immobilized on different functionalized macroporous epoxy acrylic Sepabead resins (Table 2). Four different supports were used; commercially available ones activated with epoxide, amino or butyl groups and one tailor-made functionalized with phenylboronic acid by modification of epoxy Sepabeads with 4-aminophenyl boronic acid as previously described [27].

The Ru catalyst offered (1 mg) was rapidly immobilized on the amino-Sepabeads, whereas longer times of incubation were necessary in the other three cases with a final immobilization yield of 50%–60% (Table 2). The resins free of organometallic compound were tested and no metathesis conversion was observed in any case. However, Ru catalyst was inactive and no metathesis conversion was observed. This is related to the previous effect of amines in the formation of adduct. In this way, the organometallic could react with the amino group by covalent interaction despite being completely inactive [28]. The same result was found after its adsorption on butyl-Sepabeads, however the immobilization on epoxy-Sepabeads conversed an active heterogeneous catalyst, producing **2** in 53% conversion after 24 h. Also, Ru catalyst immobilized on functionalized phenylboronic acid support catalyzed the RCM with 50% conversion after 96 h. One recycle of the boronic acid-organometallic catalyst was performed, maintaining all the initial activity. Definitely, the reaction of the Ru catalyst immobilized on supports material was lower compared to the homogeneous version, but similar to recent published results [29].

In this way, a first Ru catalyst–enzyme conjugate was prepared by incubating Hoveyda–Grubbs second-generation catalyst with lipase of *C. antarctica* (CAL-B) immobilized on butyl-Sepabeads. However, the heterogeneous organometallic biocatalyst was inactive. These results could demonstrate that the Ru catalyst was directly fully absorbed on the butyl-groups on the support and no effect of the immobilized enzyme was detected.

The differences in functional groups on the resin showed that possible adducts between Ru catalyst with epoxides [30] were more effective than with butyl groups (higher hydrophobic degree).

Considering the results obtained in the immobilization, the preparation of heterogeneous catalyst without the presence of a support material was developed. First, cross-linked enzyme aggregates (CLEA’s) of enzymes were prepared. A novel enzyme–Ru catalyst conjugate was prepared by preparation of the enzyme CLEA, adding Hoveyda–Grubbs second-generation catalyst in the solution. The method consists of the precipitation of the enzyme and a crosslinking process afterwards. Several well-described strategies determine the use of dimethoxyethane (DME) as an excellent solvent as a precipitating agent and glutaraldehyde as a crosslinking agent [31].

The stability of the Grubbs catalysts in the presence of glutaraldehyde and DME was evaluated in the metathesis reaction of **1** and the activity was not so much affected.

A variety of CLEAs of different proteins were prepared using different protocols, changing the solvent as a precipitant, an amount of protein, an amount of water or co-solvent, or the presence of different additives (data not shown). In all cases, CLEAs with and without the Ru catalyst were prepared. However, only in two cases was metathesis conversion observed. In both cases, the lipase of *C. antarctica* B was used as protein (Table 3). In these conditions, when 1 mg/mL of enzyme solution was used, the formed enzyme–Ru catalyst, CLEA, was active on the metathesis reaction, producing a 14% conversion of **1** after 1 h reaction. Using 5 mg/mL of enzyme, the resulted CLEA barely catalyzed the reaction, with 2% of product in 1 h in THF. The use of CAL-B–CLEA prepared without an organometallic molecule did not catalyze the reaction. Also, no hydrolyzed product was found using this conjugate. However, the low conversion obtained for these CLEAs is caused by the small amount of Ru catalyst present on the solid; less than 10% of the Ru catalyst offered was retained in the heterogeneous catalyst. This could be due to the fast precipitation of the protein which is not enough time for the enzyme–Ru complex interaction.

Therefore, this methodology generated a heterogeneous catalyst but with lower activity compared with the homogeneous one.

A second approach used was the entrapment system between the Hoveyda–Grubbs second-generation catalyst and the lipase by the sol–gel preparation. This strategy refers to a process where the enzyme is mixed with sol–gel solution, followed by a gelation process under the influence of pH and the aging process [32]. In the sol–gel method, an inert gel network with pore sizes is built by chemical condensation around each enzyme macromolecule, an important difference with the CLEAs strategy where an aggregated structure by covalent chemical modification is formed. Sol–gel organic/inorganic matrices were prepared using tetra-ethyl orthosilicate (TEOS) as a precursor and formic acid to catalyze the hydrolysis and condensation of silicon molecules and ethanol as a solvent. The sol–gel CAL-B without Ru catalyst were previously obtained after 24 h whereas the corresponding sol–gel Ru without protein was finally obtained after 72 h incubation. This latter was repeated, including additives such as polymers or phenylboronic acid but no gelation was observed after 72 h. Then, the conjugation of both (CAL-B and Ru with phenylboronic acid) in a sol–gel was prepared to obtain a solid gel after 24 h.

The activity of the Hoveyda–Grubbs second-generation catalyst was almost conserved in the presence of TEOS, formic acid or ethanol (data not shown).

Thus, the different sol–gel preparations were tested as a catalyst in the metathesis reaction of **1** in THF (Table 4).

The sol–gel system of lipase or the Ru catalyst alone did not catalyze the reaction (Table 4), whereas the conjugate system Hoveyda–Grubbs second-generation catalyst-CAL-B exhibited 60% metathesis conversion in 2 h; a slight improvement compared with the results using the soluble catalyst. The catalyst was used two times more, producing 36% of **2** after 2 h in the third use (maintaining 60% activity).

**Figure 1:**
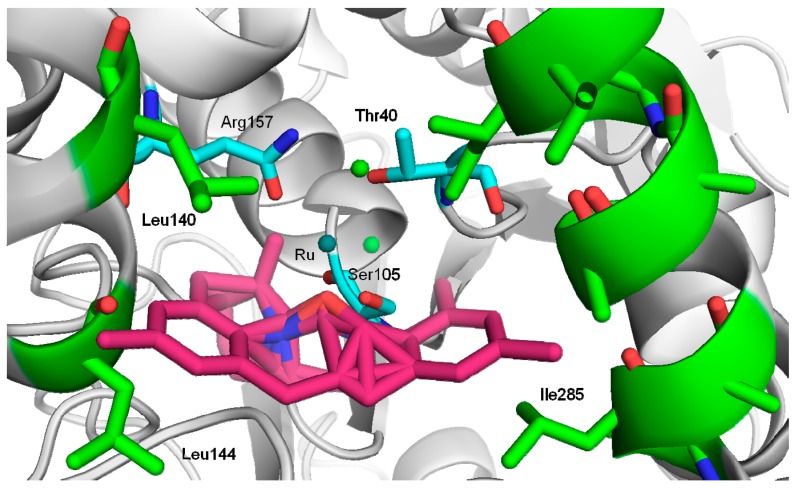
Hypothetical interaction between Hoveyda–Grubbs second-generation and CAL-B. The structure of CAL-B was obtained from the Protein Data Bank (pdb code: 1TCA) and the picture was created using Pymol v. 0.99. Hydrophobic residues (green). Hydrophilic residues (blue), Hoveyda–Grubbs second-generation (pink).

This result could demonstrate that the Ru complex is protected and stabilized by the insertion into the interior space of protein by supramolecular (non-covalent) interactions. CAL-B shows a particularly well defined oxianion cavity, with a hydrophilic area surrounding the active site (Ser105) which generates hydrogen bond interactions and a channel with high hydrophobic degree [33] where some residues such as I285, L144 and L140 are critical, for example in the location of the hydrophobic part of molecules. Therefore, considering the structure of the Grubbs catalyst, a probable hypothetic location of the catalyst could be into the channel as described in Figure 1. Combination of hydrophobic interactions of aromatic and isopropyl groups and hydrogen bond interactions of the amino groups could stabilize the conjugation with the protein.

**Figure 2:**
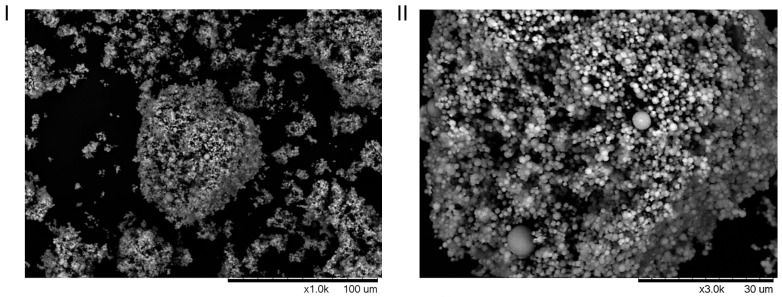
SEM images of the sol–gel Hoveyda–Grubbs second-generation catalyst-CAL-B conjugate. (**I**) Lower magnification; (**II**) Higher magnification.

In order to know the structure of the new heterogeneous Ru catalyst–lipase conjugate, Scan Electronic microscopy (SEM) was performed. In Figure 2, we can observe that a mesoporous structure with homogeneous microspheres containing lipase and Ru catalyst were formed.

One of the most interesting applications of combining enzyme and organometallic complex is performing the reaction in aqueous media.

In this way, the CAL-B–Hoveyda–Grubbs sol–gel was applied in the RCM in 2-(*N*-morpholino)ethanesulfonic acid (MES) buffer at pH 6 containing 200 mM NaCl pH 6 (Table 5). At these conditions, 50% conversion of **2** in 2 h was achieved whereas 30% was achieved using 1 mg of soluble catalyst and no conversion was observed with CAL-B sol–gel without Ru catalysts with the conjugate enzyme–Hoveyda–Grubbs in solution. After 24 h, almost full conversion was obtained (Table 5).

Thus, a new set of different heterogeneous catalysts were prepared using two different strategies. The first strategy consisted of the immobilization of CAL-B by covalent attachment on an epoxy activated Sepharose (a hydrophilic matrix) and then incubated in a solution containing Hoveyda–Grubbs second-generation catalyst (Epoxy-CAL-B–Hoveyda–Grubbs). The use of this matrix and this immobilization method was selected by considering the previous negative results with butyl-Sepabeads-lipase, avoiding the block of aromatic groups in the protein and the possible adsorption of the Grubbs into the matrix.

In the second strategy, the conjugate CAL-B and Hoveyda–Grubbs second-generation catalyst was prepared by incubation of both mixed, and then this solution was immobilized on epoxy-Sepharose (CAL-B–Hoveyda–Grubbs-Epoxy). Both hybrid catalysts were tested in the ring-closing metathesis of **1** in aqueous solution at 25 °C (Table 6).

In both cases, no adsorption of Ru catalyst was detected on the Epoxy-Sepharose support. Full conversion in the metathesis of **1** in MES buffer pH 6 containing 200 mM NaCl was observed after 2 h for both immobilized lipase–Ru catalysts and the soluble enzyme–Hoveyda–Grubbs conjugate.

In comparison of both strategy preparations, the second one was the best because similar results were achieved containing half the amount of Grubbs catalyst.

This new organometallic biocatalyst was washed with similar buffer and reused in the reaction, obtaining 80% conversion of **2** at 2 h. This process was repeated and the conversion was 60% after 2 h incubation.

## 3. Materials and Methods

### 3.1. Materials

*Candida antarctica B* lipase (CAL-B) were kindly donated by Novozymes (Bagsvaerd, Denmark). Sepabeads^®^ epoxide (SP-EC, SP-EA, SP-BU) were kindly gifted by Resindion-Mitsubishi (Binasco, Italy). Hoveyda–Grubbs second-generation catalyst, diethyldiallylmalonate, TritonX-100, tetra-ethyl orthosilicate, formic acid, glutaraldehyde, *p*-nitrophenylbutyrate (pNPP), PEG400, PEG600, PEG6000, BTFOEt, TMSOTf, 1-butyl-3-methylimidazolium tetrafluoroborate, 1-ethyl-3-methylimidazolium methyl sulphate, 1-ethyl-3-methylimidazolium tetrafluoroborate, pyridine, adenine, DEAE Dextran (2 kDa), dl-Asp–(OMe)–OMe, phenyl boric acid, NHAc–Asp–Gly–Asp–Cys–Asp–CONH_2_, triethylamine, 1,8-Diazabicyclo[5.4.0]undec-7-ene (DBU) MES buffer, Tris buffer were from Sigma Aldrich (St. Louis, MO, USA). Epoxy-Sepharose support was from General Electric Healthcare (Madrid, Spain). The scanning electron microscopy (SEM) imaging was performed on a Hitachi TM-1000 (Barcelona, Spain) microscope. The spectrophotometric analyses were run on a V-630 spectrophotometer JASCO (Madrid, Spain). HPLC spectrum P100 (Thermo Separation products) was used. Analyses were run at 25 °C using an L-7300 column oven and a UV6000LP detector (Thermo Fisher-Scientific, Waltham, MA, USA). Inductively coupled plasma optical emission spectrometry (ICP-OES) was performed on a Perkin Elmer OPTIMA 2100 DV equipment (Waltham, MA, USA). TLC analysis was performed on Merck silica gel 60 F254 (Madrid, Spain).

### 3.2. Protein Determination by Bradford Method

The Bradford Calibration was carried out as follows. Starting with 2 mg/mL Bovine Albumin Serum, six dilutions were made up with Deionized water. These dilutions were 1, 0.8, 0.6, 0.4, 0.2 and 0.1 mg/mL. An amount of 20 µL of these concentrations were added to 980 µL of Bradford Solution. These solutions were allowed to sit for ten minutes and after this ten-minute period, a UV-Visible spectrum of the solutions was taken. Using the λ_max_ which is 595 nm, the calibration plot was made. From this calibration plot, the concentration of a protein within this range can be determined accurately. If the absorbance at the λ_max_ for a given protein is too high, simply dilute the protein and rerun the spectrum. Repeat until a value between 0.2 and 0.6 is obtained for the most accurate concentration determination.

### 3.3. Determination of Ruthenium Concentration

**Figure 3:**
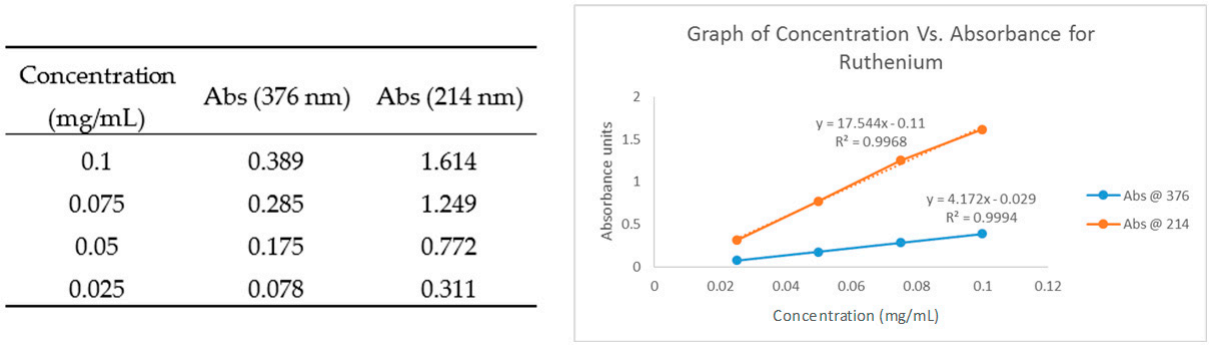
Hoveyda–Grubbs Second-Generation Calibration Curve.

To determine the concentration of ruthenium in the homogeneous catalyst, two calibration plots were made up. For each plot, five dilutions were made. These dilutions were as follows: 0.1, 0.075, 0.05, 0.025 and 0.01 mg/mL. These solutions were made from adding 1 mg of Hoveyda–Grubbs second-generation catalyst to 1 mL of DMF. A UV-Visible spectrum was taken for the above concentrations and the calibration plots were drawn. The plots will give an accurate concentration for ruthenium catalyst (Hoveyda–Grubbs second-generation). To determine the concentration of ruthenium in a solution of homogenous catalyst 1mL of the supernatant was taken and the ruthenium in the supernatant is equal to the ruthenium not in the catalyst. Total ruthenium − Ruthenium in supernatant = Ruthenium in homogenous catalyst (Figure 3). The results were confirmed by ICP-OES experiments.

### 3.4. Ring-Closing Metathesis of Diethyldiallylmalonate *(**1**)* Catalyzed by Hoveyda–Grubbs Second-Generation and Derivatives in Organic Solvent

Diethyldiallylmalonate (**1**) (8 µL, 0.033 mmoles) was dissolved in 1 mL of solvent, some cases adding 5 eq of additives (0.16 mmoles) and catalyst (1 mg soluble or 10 mg immobilized, 20 mg sol–gel, ca. 0.48 mol % Ru) were added to the solution. The reaction was stirred for 25 to 120 min, depending on the case, at 25 °C. The reactions were monitored by analytical thin-layer chromatography (TLC) using silica gel 60 F254 pre-coated glass plates (0.25 mm thickness). Visualization was accomplished by staining in an iodine chamber. Also, samples were confirmed by GC following the conditions previously described [34].

### 3.5. Immobilization of Hoveyda–Grubbs Second-Generation on Functionalized Sepabeads Resins

Functionalized Sepabead supports (epoxy, amino, phenylboronic acid, butyl, CRL-lipase) (10 mg) were added to 2 mL of THF containing 1 mg of Hoveyda–Grubbs second-generation catalyst and incubated for 3 days (except for amino Sepabeads incubated for 2 h). During this time, the solution containing the organometallic compound was analyzed by UV to determine the amount of ruthenium catalyst immobilized on the resin. Finally, the resin was filtrated and then washed with THF two times and then left to dry overnight in air.

#### 3.5.1. Preparation of Phenyl Boronic Functionalized Sepabeads

Two grams of amino Sepabeads (EC–EA) were washed in a sintered glass funnel with water and the water was removed by vacuum. An amount of 0.33 g of amino phenylboronic acid was dissolved in 7 mL of solution of water–dioxane (80:20, *v*/*v*) at pH 8.5. Then the washed and dried support was added to the amino phenylboronic acid solution and incubated for 24 h. After that, the boronic acid support was filtered and incubated in a 0.5 M solution of Trisma base for 2 h, then filtered and washed with distilled water, acetone three times, and THF. Finally, the functionalized support was dried under vacuum filtration and stored at 4 °C.

#### 3.5.2. Preparation of CAL-B–Lipase functionalized Sepabeads

Three milliliters of commercial *Candida antarctica* lipase was dissolved in 50 mL of 25 mM sodium phosphate buffer at pH 6. One gram of butyl-Sepabeads support was added to this solution and the mixture was stirred for 3 h. After that, the resin was filtered under vacuum and washed with distilled water (5 × 50 mL). Finally, it was filtered under vacuum filtration and stored at 4 °C.

### 3.6. Cross-Linked Enzyme Aggregates (CLEA’s)

The CLEAs preparation was as follows: 0.2 mL or 1 mL of lipase (CAL-B) was added to 1 mL of buffer pH 7, Na_2_PO_4_, 5 mM. Then, 100 µL of dimethoxyethane (DME) containing 1 mg of Hoveyda–Grubbs second-generation catalyst was added to the protein solution and finally 1.9 mL of DME was added to the solution. After the solution was made as above, it was allowed to mix for 10 min before 198 µL glutaraldehyde (25% solution) was added. After that, the solution was allowed to mix for 4–5 h. Once the Ru containing CLEAs were removed from mixing they were centrifuged, then the supernatant was removed. CLEAs were washed by filling Eppendorf with 1 mL of buffer, mixing on the vortex and then centrifuging the CLEAs again. This was repeated two times, then the CLEAs were washed twice with acetone; a final wash was carried out in ether and then the CLEAs were allowed to dry in the fume hood. A variety of CLEAs were made with a selection of proteins and they all followed the same procedure.

### 3.7. Sol–gel Preparations of Hoveyda–Grubbs Second-Generation

Hoveyda–Grubbs second-generation catalyst (1 mg) was dissolved in 1 mL of ethanol. This solution was added to 0.5 mL of tetraethyl orthosilicate and 169 µL of formic acid was then added. If additives were added, they were added to the tetraethyl orthosilicate at the beginning. For example, 1 mL of CAL-B/Aspartic dextran solution was added. The resulted solutions were then incubated at 40 °C for 24 h to 72 h depending on the case. After that, the resulted solid sol–gel was washed two times with ethanol and one time with THF and then left to dry at 37 °C for 3 h. The solid (80 mg containing 1 mg of Ru catalyst) was washed with ether two times and then left to dry at 37 °C for 3 h for SEM experiments.

### 3.8. Preparation of the Different Lipase–Hoveyda–Grubbs Second-Generation Epoxy Sepharose Preparations

#### 3.8.1. Strategy 1: Preparation of Epoxy-CAL-B–Hoveyda–Grubbs

Three milliliters of commercial *Candida antarctica* lipase (around 15 mg/mL) was dissolved in 25 mL of 25 mM sodium bicarbonate buffer at pH 9 containing 0.1% Triton X-100 (*v*/*v*). One gram of epoxy-Sepharose support was added to this solution and the mixture was stirred for 24 h. After that, the resin was filtered under vacuum and incubated in 25 mL solution of 1 M glycine for 30 min to block the unmodified epoxide groups of the support.

Then, the catalyst was filtered by vacuum and incubated in 5 mL of Grubbs solution (containing 2 mg of Grubbs catalyst) for 3 h. The solution was prepared dissolving 2 mg of Grubbs catalyst in 0.5 mL of ethanol. Then, this solution was mixed with 4.5 mL of 10 mM Tris Buffer + 100 mM NaCl at pH 7. Finally, the solid was filtrated and washed two times with buffer solution (10 mM Tris Buffer + 100 mM NaCl).

#### 3.8.2. Strategy 2: Preparation of CAL-B–Hoveyda–Grubbs-Epoxy

One milligram of Grubbs catalyst was dissolved in 0.5 mL of ethanol and added to a solution prepared by adding 3 mL of commercial *Candida antarctica* lipase in 15 mL of 10 mM Tris Buffer + 100 mM NaCl at pH 8 + 100 µL of Triton X-100 10% (*v*/*v*) in water. This was incubated for 3 h. After that, one gram of epoxy-Sepharose was added and the mixture was incubated for 24 h at 25 °C. Then, the resin was filtered under vacuum and washed two times with the buffer solution (10 mM Tris Buffer + 100 mM NaCl at pH 8).

In both strategies, the epoxy-Sepharose and Ru catalyst was incubated for 24 h and no ruthenium was adsorbed to the solid support, determined by UV absorbance of the Ru complex in the solution.

The amount of Ru catalyst immobilized was determined by ICP-OES analysis of the supernatant after the immobilization. A total of 85% of the initial offered Ru catalyst was conjugated with the protein.

### 3.9. Ring-Closing Metathesis of Diethyldiallylmalonate *(**1**)* Catalyzed by Immobilized Lipase-Hoveyda–Grubbs Second-Generation Hybrids in Aqueous Media

Diethyldiallylmalonate (**1**) (5 mM) was dissolved in 0.3 mL of ethanol and this solution was added to 2 mL of buffer solution (5 mM MES + 200 mM NaCl pH 6). An amount of 0.5 g of hybrid catalyst was added to this solution. The reaction was stirred for 2 h at 25 °C.

## 4. Conclusions

Here, we describe the preparation of heterogeneous hybrid conjugate *Hoveyda–Grubbs second-generation* lipase catalyst with excellent activity in the ring-closing metathesis. The optimization conditions for the homogeneous Ru catalyst were first tested and then different strategies for heterogenous formation were used. Immobilization on functionalized resins by physical adsorption or covalent attachment, covalent crosslinking binding (CLEAs), and sol–gel technology was developed obtaining different heterogeneous hybrid catalysts in mild conditions.

Excellent heterogeneous hybrid catalyst by sol–gel entrapment of the Ru catalyst together with CAL-B–lipase at mild conditions was obtained, with good activity in the ring-closing metathesis reaction in organic solvent.

Also, a set of different immobilized lipase–Grubbs heterogeneous hybrid catalysts were prepared in aqueous media and successfully applied in the RCM in aqueous media.

Therefore, the two different heterogeneous hybrid catalysts for application in organic and aqueous media in this synthetic reaction could be promising to follow in future studies in order to find an industrial applicable catalyst (much more active, stable and reusable) in chemical processes.

## Figures and Tables

**Table 1:** Additive screening for ring-closing metathesis of **1** catalyzed by soluble Hoveyda–Grubbs second-generation catalyst ^1^.

Entry	Additives	Conversion 2 (%)	Relative Activity (%) ^3^
1	-	80 ^2^	100
3	BF_3_-Et_2_O	60	75
4	TMSOTf	50	62
5	Pyridine	32	40
6	Adenosine	44	55
7	1-butyl-3-methylimidazolium tetrafluoroborate	87	109
8	1-ethyl-3-methylimidazolium methyl sulphate	88	110
9	1-ethyl-3-methylimidazolium tetrafluoroborate	87	109
10	PEG400	90	112
11	PEG600	76	95
12	PEG6000	85	106
13	phenyl-boronic acid	100	125
14	dl–Asp–(OMe)–OMe	25	31
15	NHAc–Cys–Phe–Phe–CONH_2_	85	106
16	NHAc–Asp–Gly–Asp–Cys–Asp–CONH_2_	69	86
17	Modified with BOC–Ala–Gly–COOH	15	19

^1^ Reaction conditions: **1** (8 µL, 0.033 mmoles), tetrahydrofuran (THF) (1 mL), catalyst (1 mg), 10 eq additive, 20 min and 25 °C; ^2^ we stopped the standard reaction at 80% conversion in order to see possible improvements in the activity after addition of additives; ^3^ Relative activity is the percentage calculated considering the results of the reaction without additives as 100%.

**Table 2:** Effect of the immobilization protocol on the catalytic capacity of Hoveyda–Grubbs second catalyst.^1^

Entry	Support	Ru Loading (mg)	Time (h)	Conversion 2 (%) ^2^
1	Amino-Sepabeads	1	24	<2
2	Butyl-Sepabeads	0.49	24	<2
3	Epoxy-Sepabeads	0.5	24	53
4	Phenyl Boronic acid-Sepabeads	0.6	96	50
5	Lipase-Butyl-Sepabeads	0.54	24	<2

^1^ Reaction conditions: **1** (8 µL, 0.033 mmoles), THF (1 mL), catalyst (10 mg) and 25 °C; ^2^ Calculated by TLC and HPLC analysis.

**Table 3:** Enzyme–Hoveyda–Grubbs second-generation Cross-linked enzyme aggregates (CLEA) conjugate activity in metathesis of **1**.

Entry	Catalyst	Lipase Loading (mg)	Ru Loading (mg)	Time (h)	Conversion 2 (%)
1	Hoveyda–Grubbs	-	1	1	100
2	CLEA–CAL-B	1	0	1	0
2	CLEA–Ru–CAL-B	1	<0.1	1	10
3	CLEA–Ru–CAL-B	5	<0.1	1	2

**Table 4:** Enzyme–Hoveyda–Grubbs second-generation catalyst sol–gel conjugate in metathesis reaction of **1**.^1^

Entry	Sol–Gel Conjugate	Time (h)	Conversion 2 (%)
1	- ^2^	2	65
2	Hoveyda–Grubbs	2	<2
3	CAL-B	2	0
4	CAL-B–Hoveyda–Grubbs	2	60 (36) ^3^

^1^ Reaction conditions: **1** (8 µL, 0.033 mmoles), THF (1 mL), catalyst (20 mg) and 25 °C; ^2^ the reaction performed with the soluble catalyst (0.3 mg); ^3^ conversion after third cycle.

**Table 5:** Enzyme–Hoveyda–Grubbs second-generation catalysts in ring-closing metathesis (RCM) of **1** in 2-(*N*-morpholino)ethanesulfonic acid (MES) buffer at pH 6 and 200 mM NaCl and 25 °C ^1^.

Entry	Catalyst	Time (h)	Conversion 2 (%)
1	Hoveyda–Grubbs ^2^	2	30
2	CAL-B sol–gel	2	0
3	CAL-B–Hoveyda–Grubbs sol–gel	2 (24)	50 (90)

^1^ Reaction conditions: **1** (5 mM), 5 mM MES buffer pH 6 + 200 mM NaCl (2 mL), ethanol (0.3 mL), sol–gel CAL-B–Ru or sol–gel CAL-B (0.8 g); ^2^ the reaction performed with the soluble catalyst (1 mg).

**Table 6:** Immobilized lipase-Hoveyda–Grubbs second-generation catalyst conjugates in aqueous metathesis reaction of **1** ^1^.

Entry	Catalyst	Time (h)	Conversion 2 (%)
1	Epoxy-CAL-B–Hoveyda–Grubbs ^2^	2	100
2	CAL-B–Hoveyda–Grubbs-Epoxy ^3^	2	100 (60) ^4^

^1^ Reaction conditions: **1** (5 mM), MES buffer + 200 mM NaCl (2 mL), ethanol (0.3 mL) and 25 °C biocatalyst (0.5 g, approx. 0.5 mg Grubbs); ^2^ 5 g biocatalyst containing 0.85 mg Grubbs; ^3^ 0.5 g biocatalyst containing 0.42 mg Grubbs; ^4^ conversion in the third cycle.
